# Supplementary material for: Splice-Junction-Based Mapping of Alternative Isoforms in the Human Proteome
Source: Cell Rep. Author manuscript; Available in PMC 2020 Jan 15. (PMC6961840; doi:10.1016/j.celrep.2019.11.026)
Supplement: 2 [file NIHMS1546469-supplement-2.zip › DF1/PXD010154/Thyroid/Thyroid_18_TG_VPDSEFPVMQCLTVAQNNAPSFCPLVVLPSLTEK.pdf]

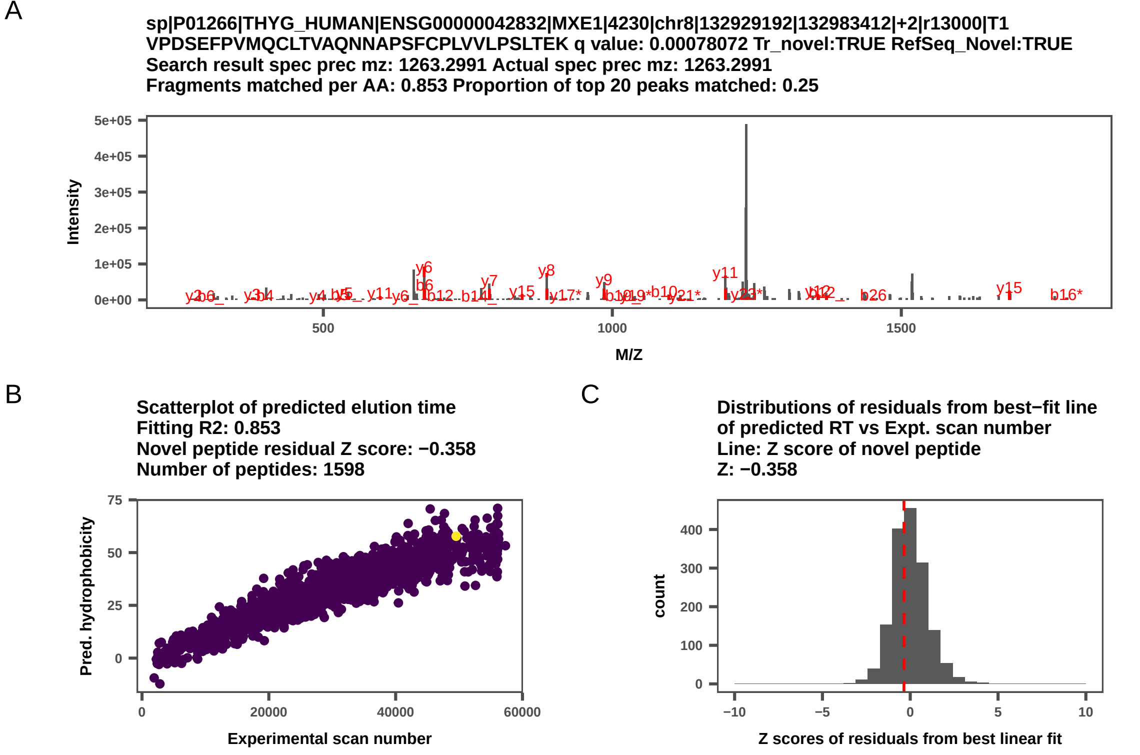
A
sp|P01266|THYG_HUMAN|ENSG00000042832|MXE1|4230|chr8|132929192|132983412|+2|r13000|T1
VPDSEFPVMQCLTVAQNNAPSFCPLVVLPSLTEK q value: 0.00078072 Tr_novel:TRUE RefSeq_Novel:TRUE
Search result spec prec mz: 1263.2991 Actual spec prec mz: 1263.2991
Fragments matched per AA: 0.853 Proportion of top 20 peaks matched: 0.25
Intensity
5e+05
4e+05
3e+05
2e+05
1e+05
0e+00
500
1000
1500
M/Z
B
Scatterplot of predicted elution time
Fitting R2: 0.853
Novel peptide residual Z score: −0.358
Number of peptides: 1598
Pred. hydrophobicity
75
50
25
0
0
20000
40000
60000
Experimental scan number
C
Distributions of residuals from best−fit line
of predicted RT vs Expt. scan number
Line: Z score of novel peptide
Z: −0.358
count
400
300
200
100
0
−10
−5
0
5
10
Z scores of residuals from best linear fit
